# Modeling the Rate- and Temperature-Dependent Behavior of Sintered Nano-Silver Paste Using a Variable-Order Fractional Model

**DOI:** 10.3390/ma18194595

**Published:** 2025-10-03

**Authors:** Qinglong Tian, Changyu Liu, Wei Cai

**Affiliations:** 1 College of Mechanical and Electrical Engineering, Hohai University, Changzhou 213200, China; tian-ql@hhu.edu.cn (Q.T.); 19033756623@163.com (C.L.); 2 College of Mechanics and Engineering Science, Hohai University, Nanjing 211100, China

**Keywords:** variable-order fractional model, coupling effects, temperature and rate dependence, sintered nano-silver paste

## Abstract

Sintered nano-silver paste is widely used in electronic packaging due to its excellent thermal and electrical conductivity. A phenomenological variable-order fractional constitutive model has been developed to characterize the evolution of its mechanical properties, incorporating dependencies on both temperature and strain rate. Based on the Weissenberg number and classical Arrhenius equation, a formulation for relaxation time with temperature and strain rate dependence has been proposed. A temperature- and rate-sensitive fractional order is introduced to capture the coupled influences of thermal and strain rate effects. Furthermore, the effects of temperature and the strain rate on the elastic modulus and relaxation time are quantitatively described through established coupling criteria. Simulation results demonstrate that the proposed model offers high accuracy and strong predictive capability. Comparisons with the classical Anand model highlight the effectiveness of the variable-order fractional model, particularly at lower temperatures.

## 1. Introduction

With the ongoing trend toward integrated, miniaturized, and high-power electronic devices, traditional soldering materials have increasingly revealed their limitations in withstanding the extreme operating temperatures and meeting the complex demands of modern electronic packaging [1]. There is a pressing need to develop alternative bonding materials with superior electrical conductivity and thermal resistance. Silver, possessing both of these desirable properties [2], emerges as a promising candidate. However, due to its high melting point, bulk silver could damage substrates during welding. To overcome this issue, silver is processed into fine powder, enabling low-temperature processing [3]. As a result, nano-silver has become a widely used bonding material in electronic packaging [4]. Given that the mechanical properties of sintered nano-silver are critical determinants of packaging reliability, a thorough investigation into its stress–strain behavior under actual service conditions is essential for accurate device lifetime assessment.

Numerous scholars have conducted experimental investigations to explore the mechanical behavior of sintered nano-silver paste [5,6,7], and have proposed and developed various models to characterize its stress–strain evolution [8,9]. Studies have shown that sintered nano-silver paste presents distinct mechanical responses under different temperatures and external loading conditions [5]. For example, under constant-strain-rate loading, sintered nano-silver paste exhibits nonlinear deformation behavior that is significantly sensitive to both temperature and loading rate. Recently, various viscoplastic models, such as the Anand model [5], Perzyna viscoplasticity [10], and the Chaboche model [11], have been applied to characterize this phenomenon. However, the complexity of its parameters hinders both comprehension and practical engineering application. Moreover, sintered nano-silver exhibits no obvious plasticity during low-temperature or high-rate deformation. Thus, developing a simplified yet accurate model is essential to reliably capture its mechanical behavior under the combined influence of temperature and deformation rate. However, such a model remains to be developed. To address these complexities, it is urgent to establish a constitutive model that adequately accounts for the influence of temperature and loading rate.

As an extension of integral calculus, fractional calculus has been successfully applied to describing various complex phenomena, including anomalous diffusion [12], power law decay [13], signal processing [14], and contact problems [15,16]. Since Scott Blair first employed fractional calculus to model the mechanical behavior of materials [17], this approach has been increasingly adopted to characterize complex deformation responses. Conventional fractional-order constitutive models often use constant order to represent material properties [18]. Extensive experiments have found that the mechanical response of many materials depends on time, loading rate, and loading history. Consequently, variable-order fractional constitutive models have been introduced to improve characterization accuracy [19,20,21]. In such models, the time-dependent variation in the fractional order reflects the material’s memory-dependent behavior. However, the existing fractional-order model for nano-silver paste fails to account for the coupling effects of temperature and strain rate.

Recently, Wang et al. [22] constructed a variable-order fractional constitutive model to capture the temperature-dependent deformation of sintered nano-silver paste. However, the above-mentioned model did not account for the coupling effects of temperature and strain rate. To address this limitation, a variable-order fractional constitutive model with explicit temperature and strain rate dependence has been developed and validated, and furthermore, its predictive accuracy has been benchmarked against the Anand model.

The rest of this paper is structured as follows: In Section 2, a variable-order fractional constitutive model is established to describe the coupling effects of temperature and strain rate under a constant strain rate. In Section 3, the proposed fractional model is successfully applied to characterizing experimental data, and is further compared with the existing model to demonstrate its superiority. In Section 4, the physical interpretations are discussed. Section 5 summarizes the conclusions.

## 2. Rate- and Temperature-Dependent Variable-Order Fractional Model

In order to characterize the one-dimensional nonlinear deformation behavior of nonlinear materials, a fractional-order constitutive model corresponding to the springpot (Scott–Blair model) was proposed [23]:

(1) σt=EθαDα0εt, 0<α<1

where *σ*(*t*) and *ε*(*t*) denote stress and strain, respectively; *E* represents the elastic modulus; *θ* refers to the relaxation time; the fractional order *α* varying between 0 and 1 is used to characterize the intermediate properties between an ideal solid and pure fluid; and the Caputo fractional derivative _0_D^α^*f*(*x*) is defined as follows [24]:

(2) Dα0fx=1Γ1−α∫0xf′τx−ταdτ, 0<α<1

where Γ(·) can be expressed as

(3) $$\Gamma \left(t\right)=\int_{0}^{\infty }e^{-\tau }\tau^{t-1}d\tau$$

Afterwards, to characterize the continuous historical dependence of properties [25,26,27], the variable fractional-order constitutive model was well developed. Experimentally, stress–strain data were predominantly acquired under constant-strain-rate conditions. This practice applies to both monotonic and cyclic tests, where the same rate is typically maintained during loading and unloading. The constitutive model, Equation (1), under the constant-strain-rate loading condition, i.e., *ε* = *ct*, can be expressed as follows [28,29]:

(4) $$\begin{array}{c} \sigma =\frac{E\theta c}{\Gamma \left(2-\alpha \right)}t^{1-\alpha },\;0<\alpha <1\\ =\frac{E{\left(\theta c\right)}^{\alpha }}{\Gamma \left(2-\alpha \right)}\varepsilon^{1-\alpha },0<\alpha <1 \end{array}$$

where *c* represents the constant strain rate. Furthermore, Equation (4) is generalized to relate the variable fractional order directly to the evolution of mechanical properties, as follows:

(5) $$\sigma \left(\varepsilon \right)=\frac{E{\left(c\theta \right)}^{\alpha \left(t\right)}}{\Gamma \left(2-\alpha \left(t\right)\right)}\varepsilon^{1-\alpha \left(t\right)},\;0<\alpha \left(t\right)<1$$

where *α*(*t*) is a continuous function, which usually appears as a linear or power law function [28,29].

The fractional-order model can describe material properties ranging from fluid-like to ideal solid-like responses. However, in conventional variable-order fractional models, the fractional order itself is defined as a function of time or strain to reflect the evolution of material properties during loading. Since the temperature and strain rate also significantly influence these mechanical properties, the fractional order must inherently correlate with both thermal and loading rate conditions—a relationship not captured by traditional formulations. To account for the coupling effects of temperature and strain rate on the mechanical properties, the classical variable-order fractional model can be further modified as follows:

(6) $$\sigma \left(\varepsilon ,\;T,\;c\right)=\frac{E\left(T,\;c\right){\left[c\theta \left(T,\;c\right)\right]}^{\alpha \left(\varepsilon ,\;T,\;c\right)}}{\Gamma \left[2-\alpha \left(\varepsilon ,\;T,\;c\right)\right]}\varepsilon^{1-\alpha \left(\varepsilon ,\;T,\;c\right)},\;0<\alpha \left(\varepsilon ,\;T,\;c\right)<1$$

where the fractional order *α* is defined as a function of the temperature, strain, and strain rate, which can be expressed as

(7) $$\alpha \left(\varepsilon ,\;T,\;c\right)=w_{1}\left(T,\;c\right)\varepsilon^{w_{2}\left(T,\;c\right)}+w_{3}\left(T,\;c\right)$$

where *w*_1_, *w*_2_, and *w*_3_ are temperature- and strain rate-dependent parameters.

As a fundamental principle, an elevated temperature promotes molecular mobility, shortening the relaxation time, while a higher strain rate inhibits relaxation, leading to a longer apparent. The temperature dependence of the relaxation time is well described by the Arrhenius equation or the Vogel–Fulcher–Tammann equation, while the strain rate dependence can be characterized by the Weissenberg number (*Wi* = *c* × *θ*). Applying logarithmic transformation to both sides of the definition of *Wi*, the relationship between ln(*θ*) and ln(*T*) can be established [28]. Inspired by the discussions mentioned above, the influence of temperature and strain rate on the relaxation time can be hypothesized and defined here as

(8) $$\ln\left[\theta \left(T,\;c\right)\right]=\theta_{r}\left(c\right)\ln\left(\frac{T}{T_{ref}}\right)+\lambda_{2}\left(c\right)$$

where the parameters *θ_r_*(*c*) and *λ*_2_(*c*) are defined as functions of the strain rate, and *T*_ref_ represents the reference temperature. The above equation can be transformed to $\theta \left(T,\;c\right)=\exp\left(\lambda_{2}\left(c\right)\right)\exp\left(\theta_{r}\left(c\right)\ln\left(\frac{T}{T_{ref}}\right)\right)$, which shares a similar form to the Arrhenius and Vogel–Fulcher–Tammann formulations at a given strain rate.

Meanwhile, it has been widely recognized that an increase in temperature causes a decrease in the elastic modulus of materials. Moreover, the elastic modulus is also obviously affected by the strain rate. Hence, in this paper, the temperature- and strain rate-dependent elastic modulus is similarly defined as follows:

(9) $$\ln\left[E\left(T,\;c\right)\right]=E_{r}\left(c\right)\ln\left(\frac{T}{T_{ref}}\right)+\lambda_{1}\left(c\right)$$

where *E_r_*(*c*) and *λ*_1_(*c*) are strain rate-dependent parameters. This equation can degenerate to the linear one under certain circumstances [20].

## 3. Application of the Proposed Variable-Order Fractional Model

The mechanical response of sintered nano-silver paste under constant-strain-rate loading is obviously dependent on the temperature and strain rate. In this section, the stress–strain relationship of sintered nano-silver paste is characterized by the proposed constitutive model to demonstrate the model’s applicability. Then, the effects of temperature and strain rate on sintered nano-silver paste are discussed. The proposed model is further compared with the famous Anand model to show its superiority.

Yu et al. [5] prepared 15 mm × 5 mm × 100 um sintered nano-silver paste and tested the mechanical properties of the sintered nano-silver paste using a dynamic mechanical analyzer (DMA-Q800) in various experiments with different temperatures (from 233.15 K and 458.15 K) and strain rates (0.001% s^−1^, 0.01% s^−1^, and 0.1% s^−1^). In this section, these experimental data are employed for further analysis.

### 3.1. Characterization at a Specific Temperature and Strain Rate

**Figure 1:**
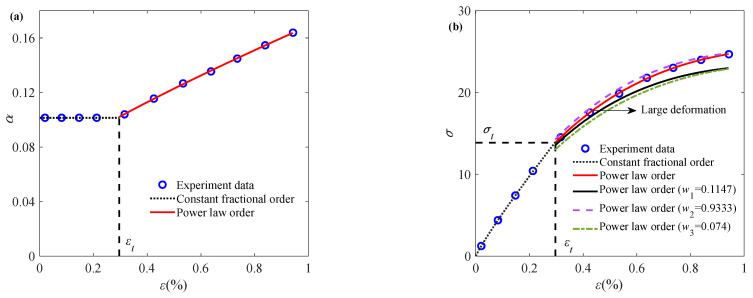
Application of fractional order constitutive model at specific temperature and strain rate: (**a**) relationship between fractional order and strain; (**b**) stress–strain relationship (experimental data cited from Ref. [5]).

In this subsection, the parameter identification procedure for the fractional-order constitutive model capturing the deformation behavior of sintered nano-silver paste is introduced here with a set of representative experimental data at a specific temperature and strain rate. Figure 1 shows the experimental data of sintered nano-silver paste stretched at a constant strain rate of 0.1% s^−1^. It can be seen from the figure that the mechanical response of sintered nano-silver paste during the tensile process is nonlinear.

To accurately characterize the relationship between the stress and strain of the sintered nano-silver paste, logarithmic transformation is applied to Equation (4):

(10) $$\ln\left[\sigma \left(\varepsilon \right)\right]=\left(1-\alpha \right)\ln\left(\varepsilon \right)+\ln\left[\frac{E{\left(c\theta \right)}^{\alpha }}{\Gamma \left(2-\alpha \right)}\right]$$

It can be seen from Equation (10) that the stress and strain relationship presents a linear relationship under the double logarithm coordinates, with a slope of 1−*α*. Therefore, the experimental data in Figure 1 are processed logarithmically. As for ln(*ε*) < ln(*ε_t_*), the relationship between ln(*ε*) and ln(*σ*) is approximately linear. The constant fractional order *α* = 0.1014 in the initial stage can be obtained by calculating the slope according to Equation (10). At this time, the constant fractional order α can better describe the experimental data.

Meanwhile, for ln(*ε*) > ln(*ε_t_*), there is a large deviation between the fitting result and the experimental data, and the fractional-order constitutive model with a constant order cannot describe this stage. Substituting the constant fractional order obtained at the initial stage into Equation (4), the corresponding elastic modulus *E* and relaxation time *θ* can be obtained, as well as the strain-dependent variable fractional order *α*(*ε*), as shown in Figure 1a. The corresponding parameter values are listed in Table 1.

From Figure 1, it can be found that when *ε* > *ε_t_*, the fractional order *α*(*ε*) is positively correlated with the strain, indicating that the resistance to deformation of the sintered nano-silver paste becomes smaller during the stretching process, and the material properties gradually become softer. The fractional order is always between 0 and 1, which is consistent with the classical fractional constitutive model frame. The stress–strain relationship of sintered nano-silver paste is described according to the fitted variable fractional order, and the relevant results are shown in Figure 1b. A comparison of the curves in Figure 1b reveals that parameters *w*_1_ and *w*_3_ are markedly more influential on the fitting results than *w*_2_. It is important to note that the symbol *ε_t_* is introduced solely for descriptive convenience, and is not utilized in the actual parameter identification and fitting procedures.

### 3.2. Temperature- and Strain Rate-Dependent Model Parameters

In the previous section, the fractional derivative model was applied to describe the stress–strain relationship of sintered nano-silver paste at a specific temperature and strain rate. However, in actual working conditions, different temperatures and strain rates will affect the mechanical properties during the interconnection of electronic packaging materials. Therefore, the effects of temperature and strain rate on the elastic modulus *E* and relaxation time *θ* are discussed in this subsection.

**Figure 2:**
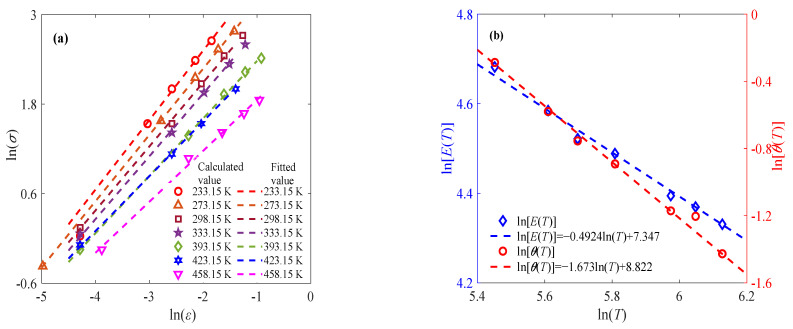
Application of fractional-order constitutive model at constant strain rate of 0.01% s^−1^: (**a**) stress–strain relationship at initial stage at different temperatures; (**b**) effects of temperature with respect to elastic modulus and relaxation time.

The stress–strain relationship of sintered nano-silver paste from 233.15 K to 458.15 K at a constant strain rate of 0.01% s^−1^ is selected for discussion [5]. Similarly to the processing method in the previous subsection, the experimental data under the double logarithmic coordinate system are processed to obtain the constant fractional order α at different temperatures in the initial stage, as shown in Figure 2a. The determined constant fractional order *α* is further substituted into the fractional order constitutive model to achieve the corresponding parameters *E* and *θ* at different temperatures. As shown in Figure 2b, the elastic modulus *E* and relaxation time *θ* decrease with increasing temperature, which is consistent with the classical theory and can be described by Equations (8) and (9).

**Figure 3:**
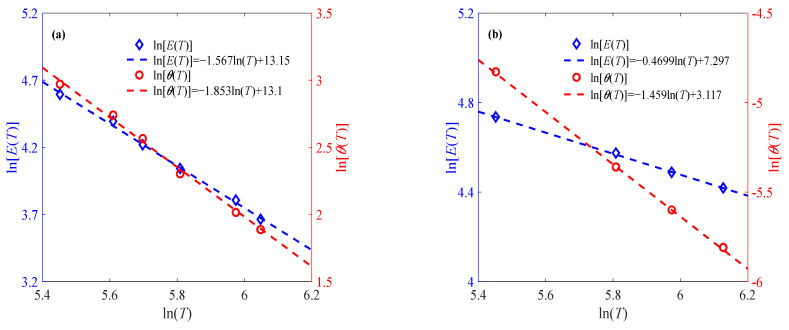
Effects of temperature on elastic modulus and relaxation time at different strain rates (**a**) 0.001% s^−1^, (**b**) 0.1% s^−1^.

**Figure 4:**
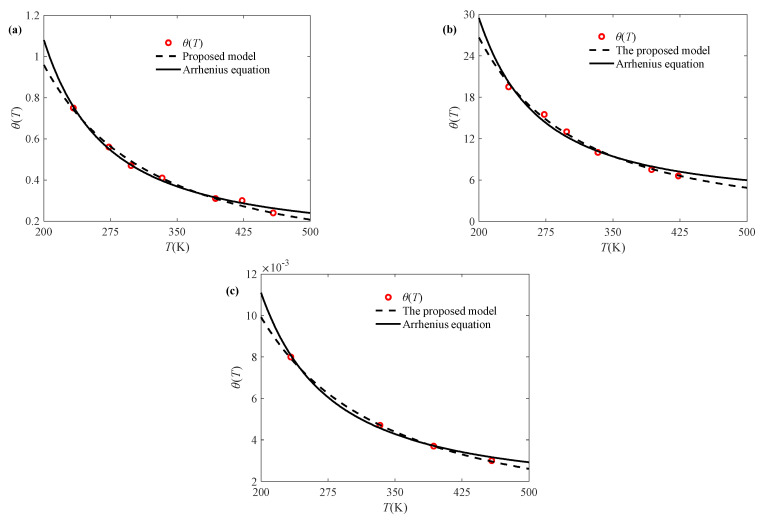
Model comparison of relaxation time at different strain rates: (**a**) c = 0.01% s^-1^, (**b**) c = 0.001% s^-1^, (**c**) c = 0.1% s^-1^.

In order to illustrate the rationality of Equations (8) and (9), another two groups of temperature-related sintered nano-silver paste data are selected for verification, as shown in Figure 3. It can be seen from Figure 3 that the established expressions of parameters and temperature can be used to represent the effect of the temperature variation trend on the elastic modulus and relaxation time. Moreover, it can also be found from Figure 2b and Figure 3 that parameters *E_r_*, *λ*_1_, *θ*_r_, and *λ*_2_ change regularly with the strain rate. As shown in Figure 4, the proposed model is compared against the well-known Arrhenius equation. The resulting *R*^2^ values in Table 2 demonstrate that the proposed model achieves comparable accuracy.

### 3.3. Coupling Effects of Temperature and Strain Rate on Deformation Characterized by Proposed Model

As discussed above, the elastic modulus/relaxation time and temperature/strain rate relationships are successfully constructed. Under such circumstances, Equation (6) is utilized to characterize the coupling effects of temperature and strain rate. Furthermore, with the obtained parameters, the constructed model is used to predict the deformation at 298.15 K and 423.15 K under a constant strain rate of 0.1% s^−1^.

**Figure 5:**
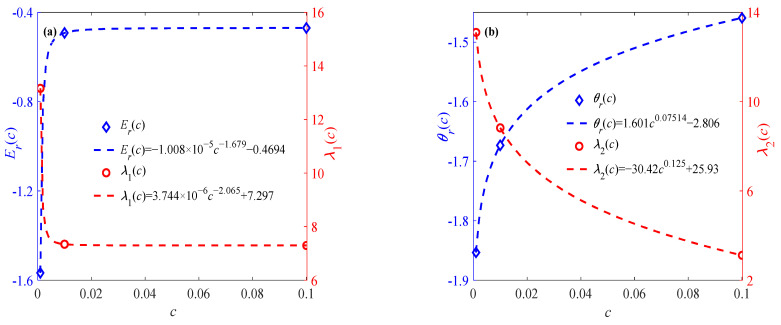
Effects of strain rate on elastic modulus and relaxation time parameters: (**a**) *E_r_*(*c*) and λ_1_(*c*), (**b**) *θ_r_*(*c*) and *λ*_2_(*c*).

Figure 5 shows the variation trends of parameters *E_r_*(*c*), λ_1_(*c*), *θ_r_*(*c*), and *λ*_2_(*c*) with respect to the strain rate, which are subsequently substituted into Equation (5) to calculate the elastic modulus *E*, relaxation time θ, and fractional order α(ε) under different temperatures and strain rates.

**Figure 6:**
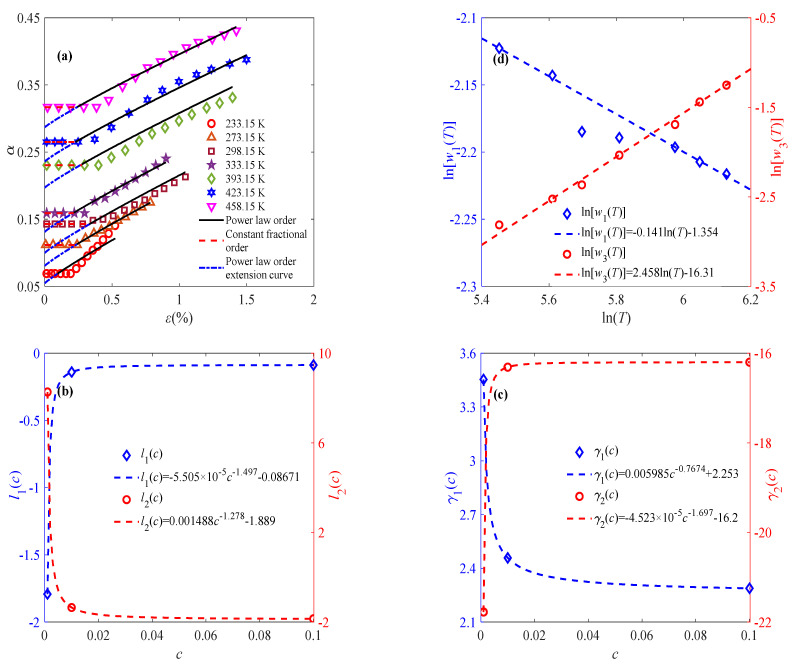
Effects of temperature and strain rate on parameters of fractional order: (**a**) relationship between fractional order and strain at different temperatures at 0.01% s^−1^; (**b**) effects of temperature on *w*_1_ and *w*_3_ at 0.01% s^−1^; (**c**) effects of strain rate on *l*_1_(*c*) and *l*_2_(*c*); and (**d**) effects of strain rate on *γ*_1_(*c*) and *γ*_2_(*c*).

Figure 6a shows the trend of variation in the fractional order of the sintered nano-silver paste under a strain of 0.01% s^−1^ and seven different temperatures. It can be found from Figure 6a that the fractional orders are power law-related to the large deformation strain, satisfying Equation (7). Hence, the parameters *w*_1_, *w*_2_, and *w*_3_ can be subsequently obtained. According to the experimental data, *w*_2_ at different temperatures is almost the same, approximated as 0.9, and is defined as

(11) $$w_{2}=\frac{1}{n}\sum_{j=1}^{n}w_{2}\left(T_{j}\right)$$

**Figure 7:**
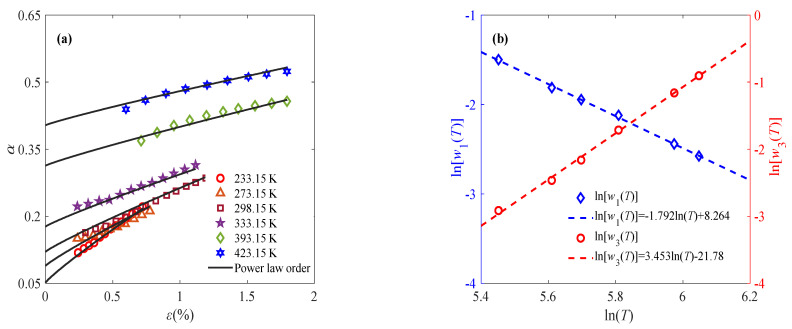
Application of fractional-order constitutive model at 0.001% s^−1^: (**a**) relationship between fractional order and strain at different temperatures; (**b**) relationship of parameters *w*_1_ and *w*_3_ with temperature.

**Figure 8:**
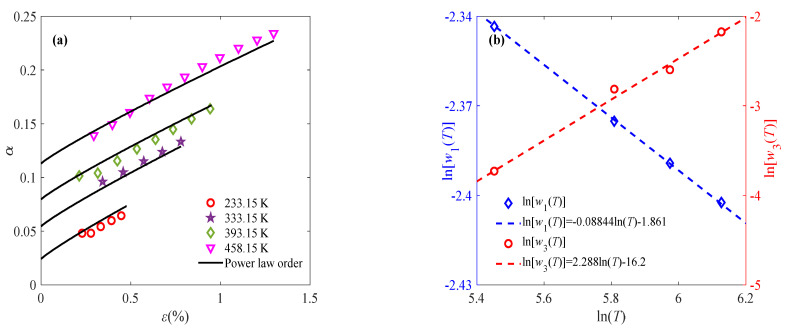
Application of fractional-order constitutive model at 0.1% s^−1^: (**a**) relationship between fractional order and strain at different temperatures; (**b**) relationship of parameters *w*_1_ and *w*_3_ with temperature.

The parameters *w*_1_(*T*, *c*) and *w*_1_(*T*, *c*) present as linear functions of the temperature, as shown in Figure 6b, which can be defined as

(12) $$\ln\left[w_{1}\left(T,\;c\right)\right]=l_{1}\left(c\right)\ln\left(T\right)+l_{2}\left(c\right)$$

(13) $$\ln\left[w_{3}\left(T,\;c\right)\right]=\gamma_{1}\left(c\right)\ln\left(T\right)+\gamma_{2}\left(c\right)$$

where the parameters *l*_1_(*c*), *l*_2_(*c*), *γ*_1_(*c*), and *γ*_2_(*c*) are defined as functions related to the strain rate, as shown in Figure 6c,d. With the obtained parameters *w*_1_, *w*_2_, and *w*_3_, the fractional order can be determined by Equation (7). The solid line in Figure 6a shows the fitting results of the viscoelastic stage, where the fractional order is composed of a constant order and a power law order. To further reduce the parameter number and uncertainty, continuous variable fractional order is used to describe the constant part. That is to say, as for *ε* > *ε_t_*, the fractional-order expression remains the same, while the constant fractional order at *ε* < *ε_t_* is approximated by the power law order. Therefore, continuous power law order can be used to characterize the complete deformation process of sintered silver paste at different temperatures and strain rates under constant-strain-rate loading. Similarly, Equation (7) is applied to describing the relationship between fractional order and strain at 0.001% s^−1^ and 0.1% s^−1^ to reflect the evolution of the mechanical properties of the material under external load, as shown in Figure 7 and Figure 8.

**Figure 9:**
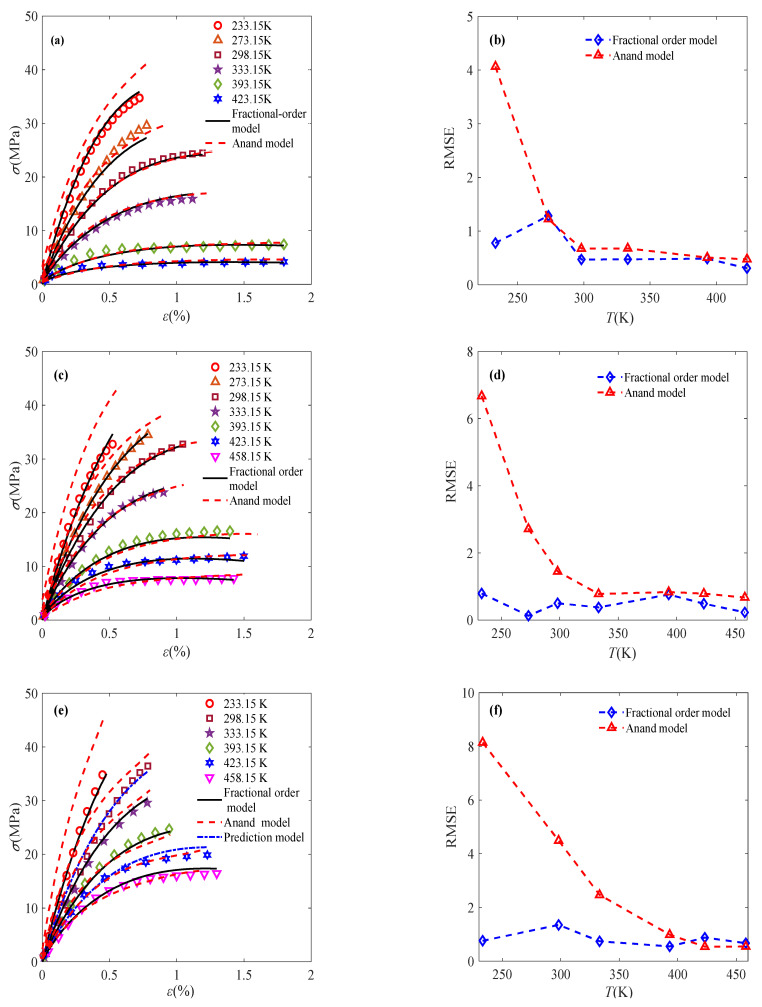
Model characterization under coupling effects of temperature and strain rate: (**a**) model characterization and comparison at 0.001% s^−1^; (**b**) RMSE comparison at 0.001% s^−1^; (**c**) model characterization and comparison at 0.01% s^−1^; (**d**) RMSE comparison at 0.01% s^−1^; (**e**) model characterization and comparison at 0.1% s^−1^; (**f**) RMSE comparison at 0.1% s^−1^ (experimental data cited from Ref. [5]).

With the help of the fractional order obtained above, the proposed fractional model is utilized to characterize the deformation of sintered nano-silver paste. The simulation results of the model are shown in Figure 9, showing good consistency with the experimental data.

In addition, the mechanical responses at 298.15 K and 423.15 K under a constant strain rate of 0.1% s^−1^ are selected in this subsection to evaluate the predictive ability of the model. The specified temperature and strain rate are substituted into the model parameter expressions to predict the material’s behavior under given conditions. The predicted results are shown in Figure 9e. It can be found from Figure 9 that the predicted results are very close to the experimental data, indicating that the fractional-order constitutive model can well predict the material’s constant-strain-rate deformation behavior considering the coupling effects of temperature and strain rate.

### 3.4. Model Comparison

At present, the Anand model is widely used in the field of microelectronics to study the deformation behavior of solder joints. Chen et al. [30] and Bai et al. [31] successfully used the Anand model to conduct mechanical analysis of different solders. Therefore, to better illustrate the superiority of our constructed fractional-order constitutive model in describing the stress–strain relationship of sintered nano-silver paste, it is further compared with the Anand model.

The Anand model can be expressed as follows [32]:

(14) $${\dot{\varepsilon }}_{p}=Ae^{\left(-\frac{Q}{RT}\right)}{\left[\sinh\left(\xi \frac{\sigma }{s}\right)\right]}^{\frac{1}{m}}$$

where ${\dot{\varepsilon }}_{p}$ stands for the rate of plastic strain, *ξ* is the stress multiplier, *T* denotes the absolute temperature, *Q* represents the activation energy, *A* stands for a constant, *m* represents the strain rate sensitivity index, *R* is the gas constant, and *s* denotes the deformation resistance.

The Anand model has been used to describe the specific deformation process of sintered nano-silver paste, and the corresponding model parameters are detailed in Ref. [5]. The comparison characterization results between the Anand model and the proposed fractional model are presented in Figure 9. It can be found from Figure 9 that the fractional derivative model has a better fitting effect. Additionally, a quantitative comparison of the *R*^2^ values at various strain rates has been reported in Table 3, Table 4 and Table 5 to strengthen the benchmarking exercise. Both the RMSE and *R*^2^ quantitively indicate that the fractional derivative model has especially better accuracy at lower temperatures. The discrepancy arises from the Anand model’s focus on high-temperature plasticity, as thermal activation softens the material. In contrast, our proposed model explicitly incorporates fractional viscoelasticity, which is critical for capturing the material’s response at low temperatures and high strain rates—a regime where the Anand model’s physical foundations are not applicable.

## 4. Physical Interpretation

**Figure 10:**
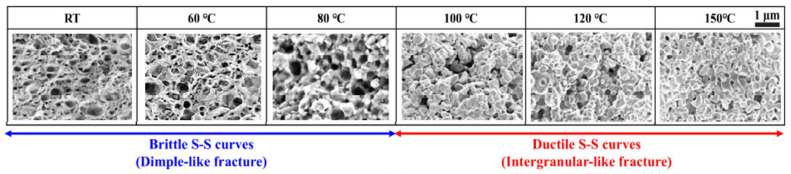
Microstructure changes at different temperatures (quoted from Ref. [33]).

Figure 9 demonstrates that the proposed fractional-order constitutive model effectively characterizes the deformation behavior of sintered nano-silver paste under constant-strain-rate conditions. The evolution of the fractional order reflects intrinsic changes in mechanical properties. As illustrated in Figure 7a, the fractional order exhibits a positive correlation of the power law with the loading process, indicating a gradual softening mechanism within the material—a behavior well-captured by the fractional calculus framework and consistent with macroscopic experimental observations. Microstructurally, the sintered nano-silver possesses a porous architecture. Upon deformation, the progressive interconnection of adjacent pores reduces the material’s resistance to external loading [33], as shown in Figure 10, which can be quantitatively represented by the increase in fractional order.

Temperature significantly influences the response of the sintered nano-silver paste. Elevated temperatures enhance ductility, leading to more pronounced deformation under identical stress levels. This is attributed to grain growth and intensified thermally activated plastic deformation [33], as shown in Figure 10. Accordingly, Figure 7a shows that the fractional order increases with temperature, validating its role in reflecting thermal softening. Furthermore, the fractional order remains bounded between 0 and 1, consistent with fractional-model constraints. Although the elastic modulus and relaxation time are treated as constants in the model for a given condition, they exhibit temperature dependence: both decrease as the temperature rises, aligning with classical viscoelastic theory.

As pointed out by Ref. [34], the strength of nano-silver materials is relatively low, primarily due to the ease of dislocation motion. To more accurately describe their mechanical behavior under high strain rates, the constitutive model developed in this work further incorporates strain rate effects. By establishing functional relationships between model parameters and the applied strain rate, quantitative description of the material’s response is achieved. These parameters exhibit clear and consistent correlations with intrinsic mechanical mechanisms—such as dislocation multiplication, grain boundary sliding, and strain rate-dependent hardening or softening behaviors. Such intrinsic connections not only validate the rationality of the parameter settings, but also demonstrate the physical reliability and applicability of the proposed constitutive framework. Thus, this model provides a robust theoretical tool for understanding and predicting the mechanical performance of nano-silver materials under various loading conditions.

## 5. Conclusions

In this paper, correlations of the loading rate and ambient temperature with model parameters (elastic modulus and relaxation time) are established. Based on the Weissenberg number and classical Arrhenius equation, a temperature- and strain rate-dependent formulation for relaxation time has been proposed. Furthermore, it has been incorporated into a phenomenological fractional model to demonstrate the coupling effects of strain rate and temperature. The fractional order is proposed to be temperature- and rate-dependent to inherently reflect thermal and rate influences on mechanical properties. The model is successfully applied to characterizing the mechanical responses of sintered silver paste, and further compared with the well-known Anand model to show the proposed model’s high accuracy and good predictivity. The specific research contents are as follows:

To characterize the constant-strain-rate deformation of sintered nano-silver paste, a variable-order fractional model is proposed that incorporates temperature and strain rate. The power law criteria are constructed to describe the coupling effects of temperature and strain rate under constant-strain-rate conditions. As the fractional orders can indicate the evolution of mechanical properties, they are also defined as a power function of the temperature and strain rate. Moreover, the model is consistent with traditional fractional theory, with fractional orders falling between 0 and 1. With the obtained parameters, the proposed fractional model can well predict deformation under various loading conditions. In addition, the accuracy of the fractional-order constitutive model is demonstrated by comparing the proposed model with the classical Anand model. However, the model’s applicability is only demonstrated for the considered dataset, and its broader validation requires future studies.

## Figures and Tables

**Table 1:** Parameter fitting results for strain rate of 0.1% s^−1^.

Parameter	*E* (MPa)	*θ* (s)	*w*_1_	*w*_2_	*w*_3_	*RMSE*	*R* ^2^
Value	89	0.0037	0.1047	0.8333	0.0643	0.0004	0.9997

**Table 2:** Comparison of *R*^2^ values for different models at different strain rates.

	c (s^−1^)	0.01%	0.001%	0.1%
*R* ^2^				
Proposed model		0.9930	0.9934	0.9998
Arrhenius equation		0.9947	0.9775	0.9965

**Table 3:** Comparison of *R*^2^ values for different models at a strain rate of 0.001%.

	*T* (K)	233.1 K	273.15 K	298.15 K	333.15 K	393.15 K	423.15 K
*R* ^2^							
Proposed model		0.9864	0.9783	0.9866	0.9923	0.9931	0.9952
Anand model		0.8391	0.9729	0.9768	0.9769	0.9889	0.9914

**Table 4:** Comparison of *R*^2^ values for different models at a strain rate of 0.01%.

	*T* (K)	233.1 K	273.15 K	298.15 K	333.15 K	393.15 K	423.15 K	458.15 K
*R* ^2^								
Proposed model		0.9875	0.9953	0.9825	0.9843	0.9826	0.9911	0.9972
Anand model		0.6831	0.8452	0.9512	0.9715	0.9723	0.9754	0.9795

**Table 5:** Comparison of *R*^2^ values for different models at a strain rate of 0.1%.

	*T* (K)	233.1 K	273.15 K	298.15 K	333.15 K	393.15 K	423.15 K	458.15 K
*R* ^2^								
Proposed model		0.9875	0.9724	0.9881	0.9904	0.9826	0.9786	0.9884
Anand model		0.5945	0.8248	0.8976	0.9896	0.9723	0.9842	0.9891

## Data Availability

The original contributions presented in this study are included in the article. Further inquiries can be directed to the corresponding author.

## Abbreviations

The following abbreviations are used in this manuscript:

RMSE	Root Mean Square Error
